# Genome‐Wide Association Study (GWAS) for Linear Body Measurements of Sussex Cattle (*Bos taurus*) at Weaning and Yearling Age in South Africa

**DOI:** 10.1002/vms3.70717

**Published:** 2025-12-11

**Authors:** Lubabalo Bila, Dikeledi Petunia Malatji, Widya Pintaka Bayu Putra, Yandisiwe Patience Sanarana, Thobela Louis Tyasi

**Affiliations:** ^1^ Department of Animal Production Potchefstroom College of Agriculture Potchefstroom South Africa; ^2^ Department of Agriculture and Animal Health College of Agriculture and Environmental Sciences University of South Africa Florida South Africa; ^3^ Research Center for Applied Zoology National Research and Innovation Agency Bogor Indonesia; ^4^ Agricultural Research Council—Animal Production Pretoria South Africa; ^5^ Department of Agricultural Economics and Animal Production School of Agricultural and Environmental Sciences University of Limpopo Sovenga South Africa

**Keywords:** ear length, exon 4, genes, Intron 1, SNP markers

## Abstract

The objective of this research was to conduct a genome‐wide association study to identify candidate genes associated with linear body measurements in South African Sussex cattle at weaning and yearling age. Overall, 96 South African Sussex animals (45 females and 51 males) were used for a GWAS with the Bovine 50K SNP BeadChip (Illumina, USA). The results indicated that three candidate genes, *RAB31*, *BIRC5* and *TEX22* were discovered as the prospective genetic markers for linear body measurements of the SA Sussex cattle at weaning age. Two candidate genes (*CFAP100* and *LOC617705*) were discovered as prospective genetic markers for linear body measurements of the SA Sussex cattle at yearling age. The two candidate genes discovered for yearling age were positioned in the following areas: *CFAP100* (3′UTR) and *LOC617705* (exon 4), respectively. A noteworthy association (*p* < 0.05) was observed between *RAB31*, *BIRC5*, *TEX22*, *CFAP100* and *LOC617705* genes for linear body measurements of the SA Sussex cattle. However, three SNPs identified at weaning age: BovineHD1100019614, BovineHD1900015483 and ARS‐BFGL‐NGS‐4967 were not located in any gene region. The findings of the current study might be used by cattle farmers to improve the linear body measurement traits of SA Sussex cattle.

## Graphical abstract

These results show that GWAS can be a useful tool for recognising novel loci and unravelling the genetic basis of complex economic traits. Sussex cattle exhibited candidate genes (*RAB31*, *BIRC5*, *TEX22*, *CFAP100* and *LOC617705*) as potential candidate genes for linear body measurement traits, which could serve as significant marker information for genome selection and contribute to the improvement of cattle breeding programs.

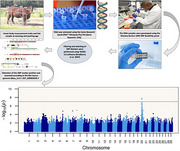

## 1 Introduction

The Sussex cattle breed is categorised by a red to dark‐red colour coat with a distinctive white tail switch of beef cattle from the South East of England (Bila et al. 2023). The Sussex cattle breed is one of the ancient pure breeds of English cattle used for beef production in the world, including countries such as South Africa (SA) and Namibia (Bila et al. 2023). Linear body measurement traits are normally involved in selection criteria for beef cattle breeding programs due to their correlation with body weight, meat production; hence, they are of great economic importance for livestock keepers (Barwick and Henzell 2005). The common growth trait used in the selection process by livestock keepers is body weight and some linear body measurement traits, which can be acquired at birth and throughout an animal's life cycle (Buzanskas et al. 2014). A genetic correlation between growth traits ranges from low to high (Baldi et al. 2010). Thereby, high genetic correlations are a result of linkage disequilibrium and pleiotropic effects of genes. However, the genes that influence the traits and the magnitude of their effects are still unknown (Terakado et al. 2017). Moreover, the use of genetic markers allows the identification of chromosome regions with huge effects on traits of our interest. According to Peters et al. (2012), there are beneficial applications, such as the understanding of the genetic structure of populations and, consequently, the application of genomic selection. The genome‐wide association study (GWAS) is a great tool for identifying loci and individual polymorphisms correlated with economically important traits in numerous species of animals (Hartati and Putra 2023). GWAS offers valuable information to advance the understanding of the genetics of complicated traits that are problematic to measure in cattle. Recently, GWAS in cattle has been used to find loci that are associated with body weight, carcass traits and meat quality traits in Nellore cattle (Santana et al. 2015; Espigolan et al. 2015; Magalhaes et al. 2016) and fatty acid composition in Simmental and Wagyu cattle of China (Zhu et al. 2017; Wang et al. 2019). In GWAS, the information of thousands of single‐nucleotide polymorphisms (SNPs) is distributed homogeneously throughout the animal's genome and is used together with the animals' phenotypes and pedigree information to make correlation analysis and recognise genes involved in the control of traits of economic importance (Bejarano et al. 2023). However, studies to determine the candidate genes for productive traits in South African Sussex cattle with GWAS are still limited (Terakado et al. 2017; Espigolan et al. 2015). Hartati et al. (2015) showed that GWAS had identified the *PLAG1* gene as the candidate gene for the calf birth weight of Ongole grade cattle. However, to the best of the authors' knowledge, there is no literature published yet on GWAS for linear body measurements of Sussex cattle (*Bos taurus*) in SA. Hence, the objective of this study was to apply the GWAS for discovering candidate genes of linear body measurements in the South African Sussex cattle at weaning and yearling age. The discoveries of this research might be beneficial to scientists and livestock keepers for instigating future genetic improvement programs.

## 2 Materials and Methods

### 2.1 Research Site, Experimental Animals and Management

The animals used in this research originated at the Huntersvlei farm in Free State Province, SA. The farm is situated in Viljoenskroon, under Fezile Dabi municipality. The research area has an astronomical location at latitude −27°20′21.05″ N and longitude 27°43′9.38″ E with an elevation of 1471 m asl; air temperature of ±30.4°C; relative humidity of 57%–89% and rainfall of ±650 mm/year (Bila et al. 2024). Overall, 96 animals (female = 45 and male = 51) were used for this study. All the animals were kept on the traditional pasture system, which allows animals to freely graze during the day with ad libitum water and regular health checks.

### 2.2 Linear Body Measurements

The data pertaining to linear body measurements and genomic analysis were taken from every animal as indicated in Table 1. Two sets of unrelated Sussex cattle animals at weaning, 55 (female = 34 and male = 21) between 6 and 8 months of age and yearling 41 (female = 11 and male = 30) between 12 and 15 months of age, were managed and used following the record kept by the farmer in this study. The linear body measurements were taken while the animal was in a standing position with its head raised and weighed on all four feet. An efficient handling facility with a crowding pen, working crush and head clamp was used for restraining the animals to lessen movement during the measuring process. In addition, the linear body measurement traits were measured in centimetres (cm) using a measuring tape, following the normal morphometric trait procedures reported by Matvieiev et al. (2025) and Bila et al. (2023).

**TABLE 1:** Linear body measurements and their description.

Traits	Description
Head length (HL)	Taken on the temple of the head to the tip of the horn
Head width (HW)	Taken as the space between the edges of the head
Ear length (EL)	Taken as the space from the point of attachment to the tip of the ear
Ear width (EW)	Taken as the distance between the middle of the top and bottom edge of the ear
Sternum height (SH)	Taken as the vertical point from the lower tip of the sternum to the ground as the animal standing
Withers height (WH)	Taken as a vertical point between the ground and the apex of the tourniquet, immediately behind the hump, on the top of the scapula
Heart girth (HG)	Taken as the circumference of the chest
Hip height (HH)	Taken as the space from the surface of a platform to the rump
Body length (BL)	Taken as the space from the highest point of shoulders to the pin bone
Rump length (RL)	Taken as the distance from the hip bone to edge of the pin bone
Rump width (RW)	Taken as the point between two tuber coxae

**FIGURE 1:**
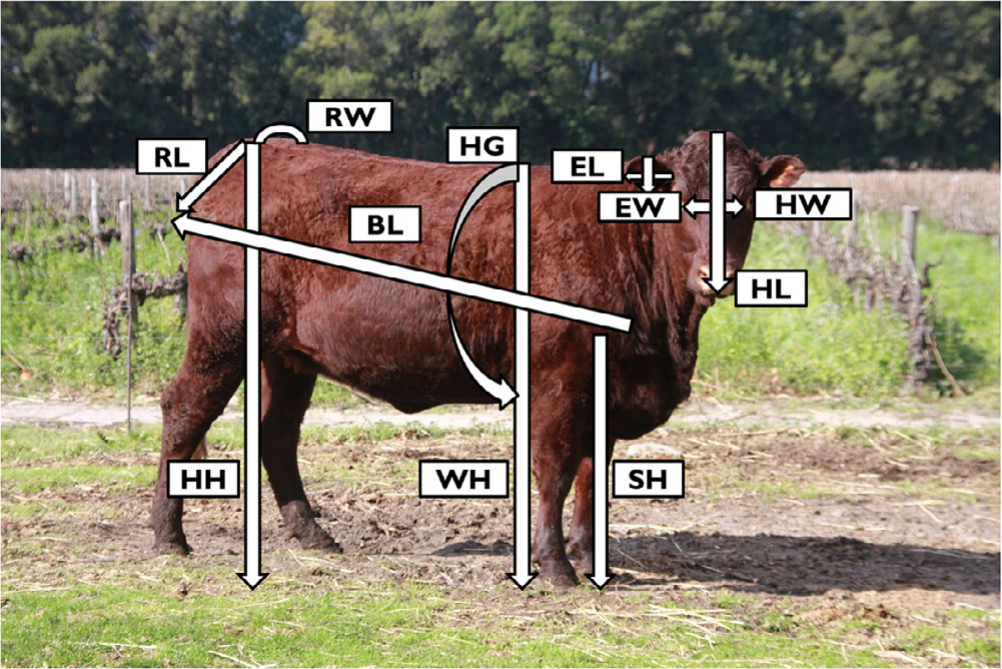
Showing linear body measurements taken during the study. BL, body length; EL, ear length; EW, ear width; HG, heart girth; HH, hip height; HL, head length; HW, head width; RL, rump length; RW, rump width; SH, sternum height; WH, withers height.

The linear body measurements such as head length (HL), head width (HW), ear length (EL), ear width (EW), sternum height (SH), withers height (WH), heart girth (HG), hip height (HH), body length (BL), rump length (RL) and rump width (RW) were taken following the guidelines defined by Hlokoe et al. (2022) using a measuring tape (Figure 1). To inhibit individual variations in the measurements, only one individual took the linear body measurements. The Statistical Package for Social Sciences (2019) version 26.0, with a probability of 5% for significance, was used to analyse the descriptive statistics of linear body measurement traits.

### 2.3 Genotyping

The tail hair samples were collected from each animal to obtain the genomic DNA material. The DNA was obtained using the Zymo Research Quick‐DNA Miniprep Plus Kit (Zymo Research, USA) following the manufacturer's protocol and quantified using the Qubit dsDNA HS assay kit (Thermo Fisher Scientific). Briefly, ∼25 hair root samples were placed in a micro‐centrifuge tube and a solution of 95 µL water, 95 µL solid tissue buffer and 10 µL proteinase K was added before mixing and incubating at 55°C for 1–3 h. Two volumes of genomic binding buffer were then added to the supernatant and mixed for 15 s. The mixture was transferred to a Zymo‐Spin llC‐XLR column in a collection tube and centrifuged at ≥ 12,000 × *g* for 1 min four times. The DNA was cleansed using a pre‐wash buffer and µL g‐DNA, then eluted using 50 µL DNA elution buffer. The eluted DNA was kept at −20°C before use. The quality and quantity of the extracted DNA were determined using 1.5% agarose gel electrophoresis (Merck) and the Qubit dsDNA HS assay kit (Thermo Fisher Scientific). The DNA samples were genotyped using the Illumina Bovine 100K SNP BeadChip panel, consisting of 95,256 markers at NEOGEN laboratory services in Lincoln, Nebraska (Illumina).

### 2.4 Filtering and Selection of SNP Markers

Filtering and picking of SNP Markers were performed using TASSEL 5.0 software (Bradbury et al. 2007). Genotype samples with less than 90% call rates were removed from the analysis. Non‐autosomal SNPs, including those with unidentified positions, were removed. SNPs with minor allele frequency value (MAF < 0.05), call rate (< 95%) and Hardy–Weinberg equilibrium (*p* < 10^−6^) were not considered further for the analysis. A total of 82,568 autosomal SNP markers (BTA1–BTA29) were used for the analysis to obtain the candidate genes. In addition, the quantile–quantile (Q–Q) and Manhattan plots were computed with the GLM statistics method to select the associated traits and the best SNP markers, respectively. In this study, a threshold value of −log_10_ *P* ^5^ was adopted to select the best candidate SNP markers that can be correlated with linear body measurements of the animal. Bonferroni was used for the significance threshold.

### 2.5 Gene Annotation and Data Analysis

Revealing of the SNP marker location was assessed according to the *B. taurus* genome (Btau_5.0.1: GCF_000003205.7) that is available on the GenBank database (https://www.ncbi.nlm.nih.gov). The genetic diversity parameters, such as genotype frequency, allele frequency, observed heterozygosity (*H*_o_), expected heterozygosity (*H*_e_), polymorphic informative content (PIC), number of effective allele (*n*_e_) and chi‐square (*χ* ^2^) values were predicted in the selected SNP (Nei and Kumar 2000). The analysis of variance (ANOVA) was computed to determine the correlation between SNP markers and linear body measurements of animals using the mathematical formula from Falconer and Mackay (1989) as follows:

$$Y_{ijk}=\mu +\alpha_{i}+\beta_{j}+\varepsilon_{ijk}$$

where *Y_ijk_* is the spotted parameter (linear body measurement traits), *μ* is the common mean, *α_i_* is the effect of sex, *β_j_* is the effect of genotype and *ε_ijk_* is the residual error.

The data correction was performed to reduce the sex effect and calculated using mathematical formulas from Searle (1960) as follows:

$$\text{CF}=X_{M}/X_{F}$$

$${\text{BM}}_{C}=X_{F}\times \text{CF}$$

where CF is the correction factor, BM_C_ is the corrected body measurements for females, *X*_M_ is the average body measurements of males and *X*_F_ is the average body weight of females.

## 3 Results

The descriptive statistics for all measured linear body measurements, by sex (male and female) at weaning age, are expressed as the mean ± standard deviation and are reported in Table 2. The results showed the male weaner animals had higher mean numeric values for the measured traits, except for rump width.

**TABLE 2:** Descriptive statistics (mean ± standard deviation), minimum and maximum for linear body measurement traits at weaning age.

	Males (*n* = 21)			Females (*n* = 34)		
Traits	Mean ± SD	Minimum	Maximum	Mean ± SD	Minimum	Maximum
HL (cm)	41.57 ± 2.42	37	46	39.41 ± 2.05	35	43
HW (cm)	13.05 ± 0.05	11	16	12.62 ± 0.82	11	14
EL (cm)	14.05 ± 0.81	13	15	13.65 ± 0.98	12	17
EW (cm)	9.62 ± 1.53	8	15	9.76 ± 0.61	8	11
SH (cm)	59.14 ± 2.87	51	65	58.41 ± 3.03	52	64
WH (cm)	110.81 ± 2.91	104	119	105.12 ± 5.08	95	113
HG(cm)	151.81 ± 17.64	82	169	148.74 ± 8.55	131	164
HH (cm)	115.81 ± 4.61	110	125	112.35 ± 6.18	97	122
BL (cm)	125.71 ± 6.28	116	137	117.74 ± 9.92	92	135
RL (cm)	39.90 ± 4.16	31	47	39.56 ± 2.55	31	44
RW (cm)	32.48 ± 3.56	23	39	32.50 ± 3.52	24	38

Abbreviations: BL, body length; cm, centimetre; EL, ear length; EW, ear width; HG, heart girth; HH, hip height; HL, head length; HW, head width; RL, rump length; RW, rump width; SH, sternum height; WH, withers height.

The descriptive statistics for all measured linear body measurements, by sex (male and female) at yearling age, are expressed as the mean ± standard deviation and are reported in Table 3. The results showed the male weaner animals had higher mean numeric values for the measured traits.

**TABLE 3:** Descriptive statistics (mean ± standard deviation), minimum and maximum for linear body measurement traits at yearling age.

	Males (*n* = 30)			Females (*n* = 11)		
Traits	Mean ± SD	Minimum	Maximum	Mean ± SD	Minimum	Maximum
HL (cm)	45.10 ± 2.56	38	49	41.18 ± 1.60	38	44
HW (cm)	21.07 ± 1.31	18	23	19.09 ± 1.64	17	22
EL (cm)	14.53 ± 0.94	13	17	14.00 ± 1.00	13	15
EW (cm)	10.23 ± 0.63	9	12	10.09 ± 0.54	9	11
SH (cm)	70.67 ± 2.43	66	78	68.18 ± 1.78	65	71
WH (cm)	129.57 ± 6.08	114	140	121.18 ± 2.09	118	125
HG(cm)	183.83 ± 15.40	126	202	166.91 ± 7.11	150	177
HH (cm)	136.07 ± 4.86	124	145	131.09 ± 3.65	126	140
BL (cm)	164.57 ± 11.60	138	183	144.64 ± 7.28	138	165
RL (cm)	49.00 ± 2.94	43	54	42.82 ± 2.09	39	46
RW (cm)	43.27 ± 2.26	34	49	39.27 ± 2.14	36	42

Abbreviations: BL, body length; cm, centimetre; EL, ear length; EW, ear width; HG, heart girth; HH, hip height; HL, head length; HW, head width; RL, rump length; RW, rump width; SH, sternum height; WH, withers height.

**FIGURE 2:**
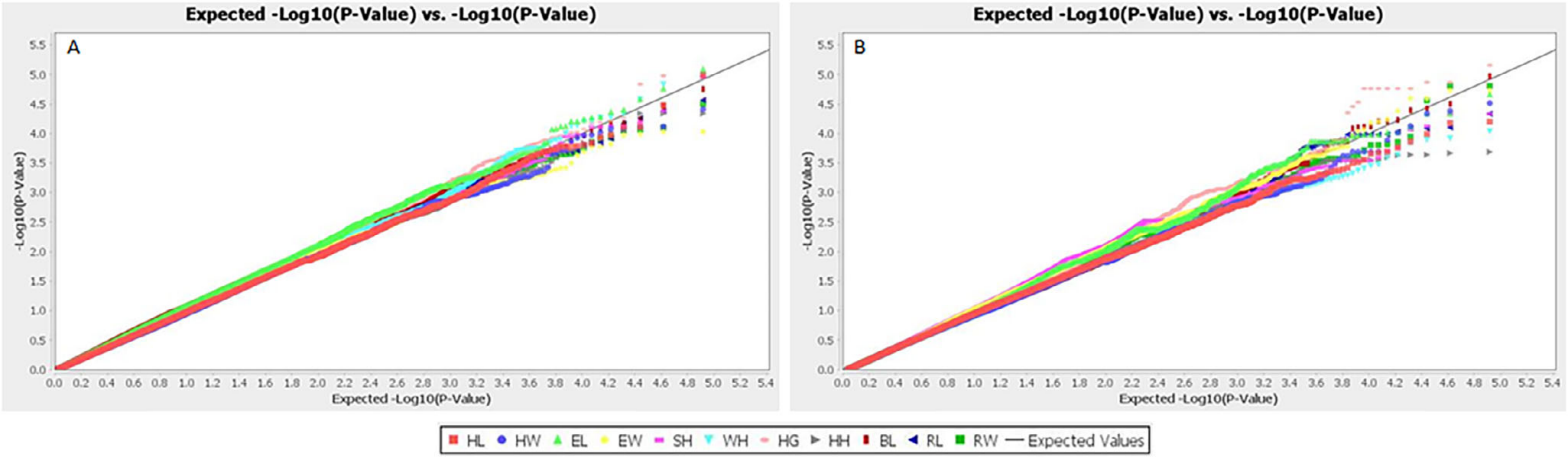
Q–Q plots for linear body measurement traits of Sussex cattle at weaning (A) and yearling (B) groups with significance thresholds indicated at −log_10_ (*p* > 5.0 × 10^−5^).

In general, the Q–Q plot indicated that five linear body measurement traits (EL, HG, HL, SH and WH) are spread above the threshold line, while six linear body measurement (HW, EW, HH, BL, RL and RW) traits are spread under the threshold line for the weaning age group (Figure 2A). On the other hand, the Q–Q plot showed that two linear body measurement (HG and BL) traits were spread under the threshold line for the yearling age group (Figure 2B).

**FIGURE 3:**
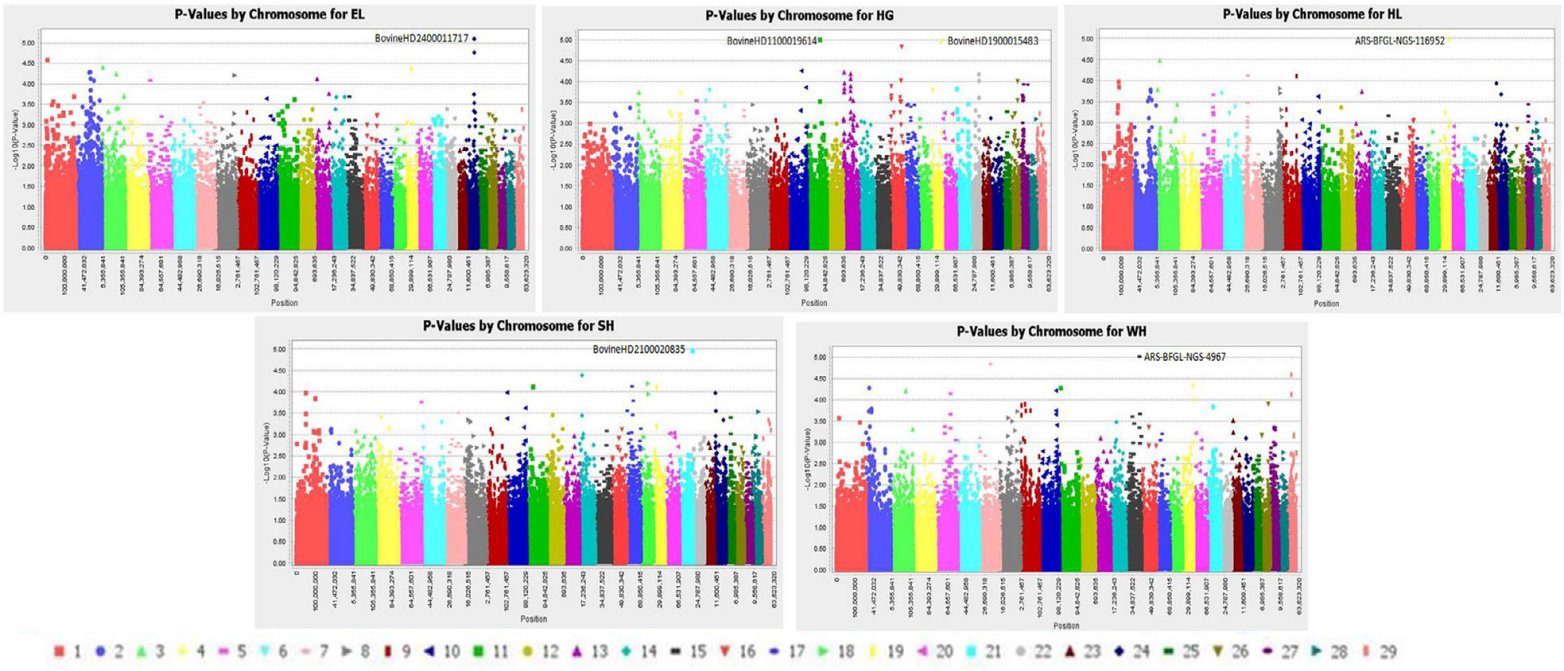
Detection of selected SNP markers (−log10^5^) in five linear body measurement traits of Sussex cattle at weaning age, that is, ear length (EL), heart girth (HG), head length (HL), sternum height (SH) and withers height (WH) in the autosomal chromosome regions (colourous dots).

**FIGURE 4:**
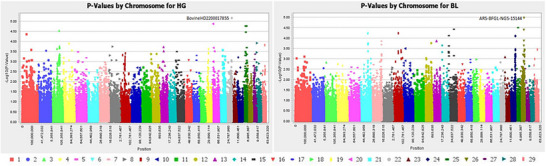
Detection of selected SNP markers (−log10^5^) in the two linear body measurement traits of Sussex cattle at yearling age, that is, heart girth (HG) and body length (BL) in the autosomal chromosome regions (colourous dots).

Figure 3 denotes the visualisation findings of the location of statistically significant polymorphic sites across 29 chromosomes for five linear body measurement traits of Sussex cattle at weaning age. Based on the Manhattan plot, six SNP markers correlated with linear body measurement (EL, HG, HG, HL, SH and WH) traits were detected on BTA 24 (BovineHD2400011717), BTA 11 (BovineHD1100019614), BTA 19 (BovineHD1900015483; ARS‐BFGL‐NGS‐116952), BTA 21 (BovineHD2100020835) and BTA 15 (ARS‐BFGL‐NGS‐4967) (Figure 3). Only three candidate genes: *RAB31* (Intron 5), *BIRC5* (Intron 1) and *TEX22* (Intron 1), significantly associated with some linear body measurement (EL, HL and SH) traits were identified on different gene positions (41,786,924–41,868,539, 53,940,674–53,947,960 and 69,685,620–69,700,788), respectively, as shown in Table 2. All the SNPs detected had *p* values ranging from 1.00E−5–9.70E−6 and MAF ranging from 0.07 to 0.42. Moreover, Figure 4 depicts the visualisation findings of the location of statistically significant polymorphic sites across 29 chromosomes for two linear body measurement traits of Sussex cattle at yearling age. Based on the Manhattan plot, two SNP markers associated with linear body measurement (HG and BL) traits were detected on BTA 22 (BovineHD2200017855) and BTA 26 (ARS‐BFGL‐NGS‐15144) (Figure 4) at yearling age. Only two candidate genes, *CFAP100* (3′UTR) and *LOC617705* (Exon 4), significantly correlated with some linear body measurement (HG and BL) traits were identified on different gene positions (60,650,677–60,702,690 and 42,607,465–42,690,008), respectively, as shown in Table 4. All the SNPs detected had p values ranging from 1.00E−5–9.70E−6 and MAF ranging from 0.07 to 0.42 for both weaning and yearling age of Sussex cattle.

**TABLE 4:** Detection of selected SNP markers and candidate genes for some linear body measurements in Sussex cattle.

Group	SNP marker	BTA	Trait	*p*	Position	MAF	Gene^a^	Region	Gene position
Weaning	BovineHD2400011717	24	EL	8.12E−6	41,855,725	0.20	*RAB31*	Intron 5	41,786,924–41,868,539
	BovineHD1100019614	11	HG	1.00E−5	69,170,364	0.27	—	—	—
	BovineHD1900015483	19	HG	1.04E−5	54,437,215	0.20	—	—	—
	ARS‐BFGL‐NGS‐116952	19	HL	1.02E−5	53,942,084	0.07	*BIRC5*	Intron 1	53,940,674–53,947,960
	BovineHD2100020835	21	SH	1.10E−5	69,697,367	0.27	*TEX22*	Intron 1	69,685,620 – 69,700,788
	ARS‐BFGL‐NGS‐4967	15	WH	9.70E−6	73,018,714	0.42	—	—	—
Yearling	BovineHD2200017855	22	HG	6.94E−6	60,700,520	0.28	*CFAP100*	3′UTR	60,650,677–60,702,690
	ARS‐BFGL‐NGS‐15144	26	BL	1.07E−5	42,610,754	0.35	*LOC617705*	Exon 4	42,607,465–42,690,008

^a^ AssemblyARS‐UCD2.0 (GCF_002263795.3).

Abbreviations: BTA, *Bos taurus* autosome; EL, ear length; HG, heart girth; HL, head length; MAF, minimum allele frequency; SH, sternum height; WH, withers height.

The PIC value in the *RAB31*, *BIRC5*, *TEX22*, *CFAP100* and *LOC617705* genes ranged from 0.07 (*moderate*) and 0.37 (*high*), respectively, as shown in Table 5. Despite this, the genetic diversity in all five genes was shown to be under the genetic equilibrium. Moreover, the number of effective alleles of *RAB31*, *BIRC5*, *TEX22*, *CFAP100* and *LOC617705* genes was 1.20, 1.08, 1.98, 1.94 and 1.47, respectively, as shown in Table 5. On the other hand, all the detected genes had a significant effect, with *CFAP100* having the highest effect of 3.35, while *BIRC5* had the lowest effect of 0.08.

**TABLE 5:** Genetic diversity in the candidate genes for linear body measurement traits in Sussex cattle.

Gene	Genotype	Allele frequency	*H*_o_	*H*_e_	*n*_e_	PIC	*χ* ^2^
*RAB31*	CC = 45 (0.82)	C = 0.91	0.18	0.16	1.20	0.15	0.55^a^
	CT = 10 (0.18)	T = 0.01					
*BIRC5*	GG = 51 (0.93)	G = 0.96	0.07	0.07	1.08	0.07	0.08^a^
	GT = 4 (0.07)	T = 0.04					
*TEX22*	CC = 15 (0.27)	C = 0.56	0.56	0.49	1.98	0.37	1.09^a^
	CT = 31 (0.56)	T = 0.44					
	TT = 9 (0.16)						
*CFAP100*	CC = 4 (0.10)	C = 0.41	0.62	0.48	1.94	0.37	3.35^a^
	CA = 25 (0.63)	A = 0.59					
	AA = 11 (0.28)						
*LOC617705*	TT = 25 (0.63)	T = 0.80	0.35	0.32	1.47	0.27	0.35^a^
	TG = 14 (0.35)	G = 0.20					
	GG = 1 (0.03)						

Abbreviations: *H*_e_, expected heterozygosity; *H*_o_, observed heterozygosity; *n*_e_, number of effective allele; PIC, polymorphic informative content; *χ* ^2^, chi square.

^a^ Under in genetic equilibrium.

Table 6 shows the effect of gene polymorphisms on some linear body measurements in Sussex cattle at weaning and yearling age. Our results show that two genes (*RAB31* and *BIRC5*) detected at weaning age were insignificant (*p* > 0.05) associated with EL and HL. On the other hand, these findings show that two genes (*CFAP100* and *LOC617705*) detected at yearling age were significantly (*p* < 0.05) correlated with HG and BL.

**TABLE 6:** Effect of gene polymorphisms with some linear body measurement traits in Sussex cattle.

Group	Gene	Related trait	Genotype	Value (cm)
Weaning	*RAB31*	Ear length	CC	13.66 ± 0.94
			CT	14.36 ± 0.67
	*BIRC5*	Head length	GG	39.94 ± 2.15
			GT	44.00 ± 2.83
	*TEX22*	Sternum height	CC	56.40 ± 2.77a
			CT	59.64 ± 2.59b
			TT	59.22 ± 2.63b
Yearling	*CFAP100*		CC	153.25 ± 20.25a
		Heart girth	CA	180.80 ± 12.40b
			AA	184.91 ± 12.75a
	*LOC617705*	Body length	TT	165.80 ± 11.69a
			TG	149.71 ± 10.76b
			GG	142.00 ± 0.00

*Note*: a and b superscript in the similar row differ significantly (*p* < 0.05).

## 4 Discussion

In general, animal weight and growth traits are considered income drivers in livestock production systems (Yu et al. 2023). Linear body measurement traits, such as the body weight and body size of beef cattle, are vital phenotypic data that are crucial to investigating the appearance and production performance of cattle types. The linear body measurement traits of beef cattle are co‐regulated by several genes, which are difficult to find. The GWAS is a good tool for screening for dominant genes correlated with linear body measurement traits in animal species such as pigs (Yu et al. 2023; Wu et al. 2019), sheep (Rovadoscki et al. 2018) and beef cattle (de Las Heras‐Saldana et al. 2020). In the study of a commercial broiler chicken population, Mebratie et al. (2019) performed GWAS using the mixed linear model (MLM) approach and found that using models that account for the population structure may lower bias and increase accuracy of the predicted SNP effects in the association analysis. In this study, numerous SNPs and candidate genes were discovered among the five linear body measurement traits of SA Sussex cattle, including weaning and yearling age. Yu et al. (2023) narrated that body length and chest circumference had the highest significant effect on body weight, with phenotypic association coefficients of 0.975 and 0.962, respectively. Ren et al. (2023) revealed eight significant SNPs identified through the MLM, with 6 SNPs associated with multiple traits and 4 SNPs (Affx‐277,062,550, Affx‐257,095,832, Affx‐115,873,673 and Affx‐41,315,554) associated with body height, body length, hip height, back height, waist height and ischial tuberosity height. Furthermore, 21 candidate genes were in proximity to or within these significant SNPs. Among them, *Scarb1*, *acetoacetylCoA* synthetase and *HIVEP3* were implicated in bone formation and rarely encountered in livestock body measurement traits, emerging as potential candidate genes regulating body measurement traits in Wenshan cattle. Calderón‐Chagoya et al. (2022) stated a total of 110, 143 and 302 SNPs with the use of GWAS and chromosome‐wide association analyses (CWAS) linked to growth traits in the Simmental and Simbrah cattle breeds, respectively. Calderón‐Chagoya et al. (2022, 25) further stated the following pathways: RNA polymerase (*POLR2G* and *POLR3E*) and GABAergic synapse (*GABRR1* and *GABRR3*) for Simmental cattle and p53 signalling pathway (*BID* and *SERPINB5*) for Simbrah cattle. Moreover, Hartati and Putra (2023) attained an intergenic SNP marker at BTA17 ARS‐BFGL‐NGS‐78232 that is significantly associated with slaughter weight and carcass weight of Sumba Ongole bulls (*Bos indicus*). Reports made by Ren et al. (2023) indicate that those three genes, *Scarb1*, *AACS* and *HIVEP3*, may be specific to body measurement traits in Chinese Wenshan cattle and have high research value. Scavenger receptor type I (Scarb1) on BTA17 was related to body height, body length, hip height, back height, waist height and ischial tuberosity height, linear body measurement traits. Thereby, the *Scarb1* produced by the SCARB1 gene is the major receptor for high‐density lipoprotein (HDL). The findings of the present study are in contrast with the reports made by Yu et al. (2023), who discovered a total of 250 significant SNPs (*p* < 3.54 × 10^−6^) for genome‐wide association analysis of 14 body conformation traits in 254 Qinchuan cattle breed and annotated to 37 candidate genes. For body weight, one SNP was found on chromosome 21, an unannotated gene. For body height, 27 SNPs were discovered on chromosome 21, annotated to 29 genes, including *ASB7*, *IGF1R*, *MEF2A* and more. For back height, chromosomes 1, 17, 18, 20 and 22 showed one SNP each with no genes annotated. As for buttock height, 16 SNPs were found on 10 different chromosomes and candidate genes were not annotated. In the current study, one SNP was found on BTA24, and the candidate gene was found in Intron 5 at the weaning age for EL. Furthermore, two SNPs were found on BTA19, and the candidate genes were not annotated at the weaning age for HG. In addition, one SNP was found on BTA19, and the candidate gene was found in Intron 1 at the weaning age for HL. For WH, one SNP was found on BTA15, and the candidate gene was found in Intron 1 at weaning age. Moreover, one SNP was found on BTA21, and the candidate gene was not annotated at the weaning age for SH. The study further shows that one SNP was found on BTA22, and the candidate gene was found in the 3′UTR at yearling age for HG. Finally, for BL, one SNP was found on BTA26, and the candidate gene was found in exon four at yearling age. Thakkar et al. (2022) reported that the MAF value can be categorised into rare (< 0.05), intermediate (0.05–0.25) and > 0.25 highly polymorphic. In the current study, the MAF values in the RAB31, BIRC5, TEX22, CFAP100 and LOC617705 genes were in the category of intermediate and high, respectively. Furthermore, Nei and Kumar (2000) reported that the PIC value could be classified into low (< 0.10), moderate (0.10–0.30) and high (> 0.30). In the current study, the PIC values in the *RAB31*, *BIRC5*, *TEX22*, *CFAP100* and *LOC617705* genes were in the category of low and high, respectively. The genetic diversity in the *RAB31*, *BIRC5*, *TEX22*, *CFAP100* and *LOC617705* genes of Sussex cattle was described under the genetic equilibrium. Falconer and Mackay (1989) argue that genetic diversity in livestock can be influenced by several factors, such as migration, selection, inbreeding and crossbreeding systems.

## 5 Conclusion

This study shows that the GWAS can be a useful tool for recognising novel loci and unravelling the genetic basis of complex economic traits. Moreover, the important candidate genes and molecular markers of linear body measurement traits were selected from the genome to analyse the potential genetic mechanism of dominant traits and provide a scientific basis for breeding improvement of beef cattle. Finally, the identified candidate genes (*RAB31*, *BIRC5*, *TEX22*, *CFAP100* and *LOC617705*) can be considered as potential candidate genes for linear body measurement traits, which could serve as significant marker information for genome selection and contribute to the improvement of cattle breeding programs.

## Author Contributions

Initial idea of the study was by Lubabalo Bila, Dikeledi Petunia Malatji and Thobela Louis Tyasi. Widya Pintaka Bayu Putra analysed the data. Lubabalo Bila wrote the first draft of the manuscript. Yandisiwe Patience Sanarana, Dikeledi Petunia Malatji and Thobela Louis Tyasi revised the manuscript. All authors read and approved the final manuscript.

## Funding

The authors would like to thank the National Research Foundation (NRF) reference number (MND210415594902) for its financial support.

## Ethics Statement

The experimental practices were conducted following the University of South Africa (UNISA) Ethics code for the use of live animals in research with ethics reference number: 2022/CAES_AREC/171.

## Conflicts of Interest

The authors declare no conflicts of interest.

## Data Availability

The data that support the findings of this study are available from the corresponding author upon reasonable request.
